# Pleiotropic microRNA-21 in pulmonary remodeling: novel insights for molecular mechanism and present advancements

**DOI:** 10.1186/s13223-019-0345-2

**Published:** 2019-05-20

**Authors:** Congshan Jiang, Yuanxu Guo, Hongchuan Yu, Shemin Lu, Liesu Meng

**Affiliations:** 1 Department of Biochemistry and Molecular Biology, School of Basic Medical Sciences, Xi’an Jiaotong University Health Science Center, West Yanta Road No.76, Xi’an, Shaanxi People’s Republic of China; 2 Key Laboratory of Environment and Genes Related to Diseases (Xi’an Jiaotong University), Ministry of Education, Xi’an, Shaanxi People’s Republic of China; 3 Department of Respiratory Medicine, Xi’an Children Hospital, Xi’an, Shaanxi People’s Republic of China

**Keywords:** microRNA-21, Pleiotropic effects, Pulmonary remodeling, Target regulation

## Abstract

MicroRNA-21 (miR-21), probably one of the most studied miRNAs to date, is found pleiotropic in various biological events. Its emerging role in pulmonary remodeling has attracted extensive attention. This review summarizes the genomic information of its primary transcript and various transcriptional regulations on its promoter. In addition, the role of miR-21 in pulmonary remodeling related signaling such as transforming growth factor β (TGF-β), bone morphogenetic protein (BMP), epidermal growth factor receptor (EGFR) and Notch signaling is discussed. Various validated miR-21 target genes participate in controlling of the overactive cell accumulation, smooth muscle contraction, inflammatory stress (trigger for lung epithelium damage), extracellular matrix deposition and hypoxia-induced disorders. Moreover, we focus on its particular implication in events including inflammatory stress-driven epithelium damage, epithelial-to-mesenchymal transition (EMT), transdifferentiation of fibroblasts into myofibroblasts, hypoxia stimuli and ROS response, as well as some other pulmonary remodeling related events such as overactive fibroblast (myofibroblast) accumulation, extracellular matrix deposition, and angiogenesis. Here, we summarize the strong potential of miR-21 in pulmonary remodeling and provide novel clues for further research in this area.

## Introduction

MicroRNA-21 (miR-21) is a pleiotropic miRNA with its frequent appearance in researches concerning the promotion of cell proliferation, inflammation, angiogenesis, and immune destruction. Its significance in molecular function has been focused on the strong implication in human cancers [1], and has been so far considered as the only miRNA with overexpression pattern in lots of distinct cancers, such as glioblastoma [2], medulloblastoma [3], head and neck squamous cell carcinoma [4], esophageal squamous cell carcinoma [5], lung cancer [6], breast cancer [6], pancreatic adenocarcinoma [6], gastric cancer [6], prostate cancer [6], cholangiocarcinoma [7], cervical carcinoma [8], colorectal cancer [9], multiple myeloma [10] and leiomyomas [11]. Its targets were identified involving in various aspects of cancer progression [12] including proliferation promoting, apoptosis inhibition, invasion, angiogenesis, and chemo-resistance. There is also an emerging role of miR-21 in immune cell polarization, muscle contraction, tissue remodeling, epithelial-to-mesenchymal transition (EMT) and pathological fibrosis, which has extensively drawn our attention.

It was found that miR-21 is also strongly implicated in pulmonary inflammation in our previous study [13]. As the important consequences of pulmonary inflammation, the repair, regeneration, and remodeling are major events of the injured respiratory system. After the injury, the lung can retain normal structure and physiological function via repair or regeneration. If failed, lung remodeling will happen which is very likely to result in harmful changes [14]. Pathological lung remodeling includes stressed or injured epithelium, increased smooth muscle mass, sub-epithelial fibrosis, goblet cell and sub-mucosal gland enlargement, hyperemia with increased vascularity of sub-epithelial tissues, thickening of basement membrane and extracellular matrix deposition [15, 16]. Airway remodeling appears in various chronic pulmonary disorders such as chronic obstructive pulmonary disease (COPD), idiopathic pulmonary fibrosis and especially adult asthma [15]. So far, mounting reports indicate that it is also common and early present in at least the moderate and severe type of pediatric asthma (2–15 years old) [17–20]. These remodeling changes contribute to airway narrowing, bronchial hyper-responsiveness, airway edema, and mucus hypersecretion, leaving patients with recurrent suffering.

Plenty of evidence indicated that miR-21 might play an important role in pathological remodeling. Our review aimed to summarize the regulation potential of miR-21 in pathological pulmonary remodeling and provide novel clues for further research.

### Genomic organization of miR-21

According to the recently released information on miRBase [21], the single primary transcript containing human miR-21 (pri-miR-21) is transcribed from an intron of a protein-coding gene TMEM49 within chromosome 17q23.2. With its promoter region, the pri-miR-21 is 3433-nt long in total. Its stem-loop precursor pre-miR-21 is 72 nt, while the mature miR-21 is 22 nt. Mature miR-21 is widely and abundantly expressed in various types of tissues and cells. Current computational approach identified miR-21 promoter resides at about 900 bp upstream from the identified transcription start site (TSS). The promoter locates in the 10th intron of TMEM49, and it has several conserved binding sites for activation protein 1 (AP-1, composed of Fos and Jun family), PU.1, C/EBPα, NFI, SRF, p53 and STAT3 [22], which makes miR-21 subject to transcriptional regulation.

### Transcriptional regulation of miR-21

#### Transcriptional activation

Nuclear factor-kappa B (NF-κB) p65 subunit direct binds to pri-miR-21 promoter, which is identified by chromatin immunoprecipitation (ChIP) analysis [23] and confirmed by the luciferase reporter assay [24]. The nicotine-induced up-regulation of miR-21 and gastric cancer cell proliferation is also dependent on NF-κB signaling activation [25]. In addition, AP-1 binds and activates miR-21 promoter synergistically with PU.1 [22]. ChIP assay result validated that the constitutively activated signal transducer and activator of transcription 3 (STAT3) directly targets and activates pri-miR-21 in Sézary Syndrome (SS), a cutaneous T-cell lymphoma (CTCL) [26].

#### Transcriptional repression

miR-21 transcription is found to be repressed by NFI and C/EBPα. NFIB protein usually binds to the miR-21 promoter as a negative regulator in HL-60 human promyelocytic cells. During miR-21 independent suppression of NFIB, NFIB is swept off from the promoter, and as a miR-21 direct target protein, NFIB expression is also inhibited at translational level [22]. BCL-6 inhibits Th2-type response as a transcriptional repressor of miR-21 in T cells. The repressor BCL-6 and the activator STAT3 bind to the same site of pri-miR-21 gene promoter, but play the opposite role on its transcription [27]. The zinc finger protein GFI1, a transcriptional repressor, which is crucial for normal granulocytic differentiation, can negatively regulate miR-21 expression. Bone marrow cells from a GFI1-mutant severe congenital neutropenia (SCN) patient and *Gfi1*^−/−^ mice display overexpression of miR-21 [28].

#### Biogenesis regulation

miRNAs undergo a classical cropping and dicing process before they become mature and functional molecules [29]. Firstly, pri-miRNA is transcribed by RNA polymerase II [30], and cropped by Drosha into a ~ 60–100nt hairpin pre-miRNA [31]. This pre-miRNA is transported out of cell nucleus accompanied by Expotin-5 and Ran-GTP [32], and further processed by Dicer to produce a ~ 22 nt double-stranded RNA containing the mature and the passenger one (named miRNA*). Eventually, this mature miRNA interacts with the target mRNA and hence post-transcriptionally [33] regulate its production within special machinery called RNA-induced silencing complex (RISC). This biogenesis requires various factors to participate in every single step of processing [34]. Among them, Drosha and Dicer, as the cropping and dicing enzymes, are particularly significant. Knockdown of dicer by siRNA leads to down-regulation of miR-21, significant G_1_ arrest and more sensitivity to cisplatin in MCF-7 breast cancer cells [35]. TGF-β and BMP induce ligand-specific SMAD protein recruitment to Drosha complex and promotes pri-miR-21 transforming into pre-miR-21 [36].

**Fig. 1:**
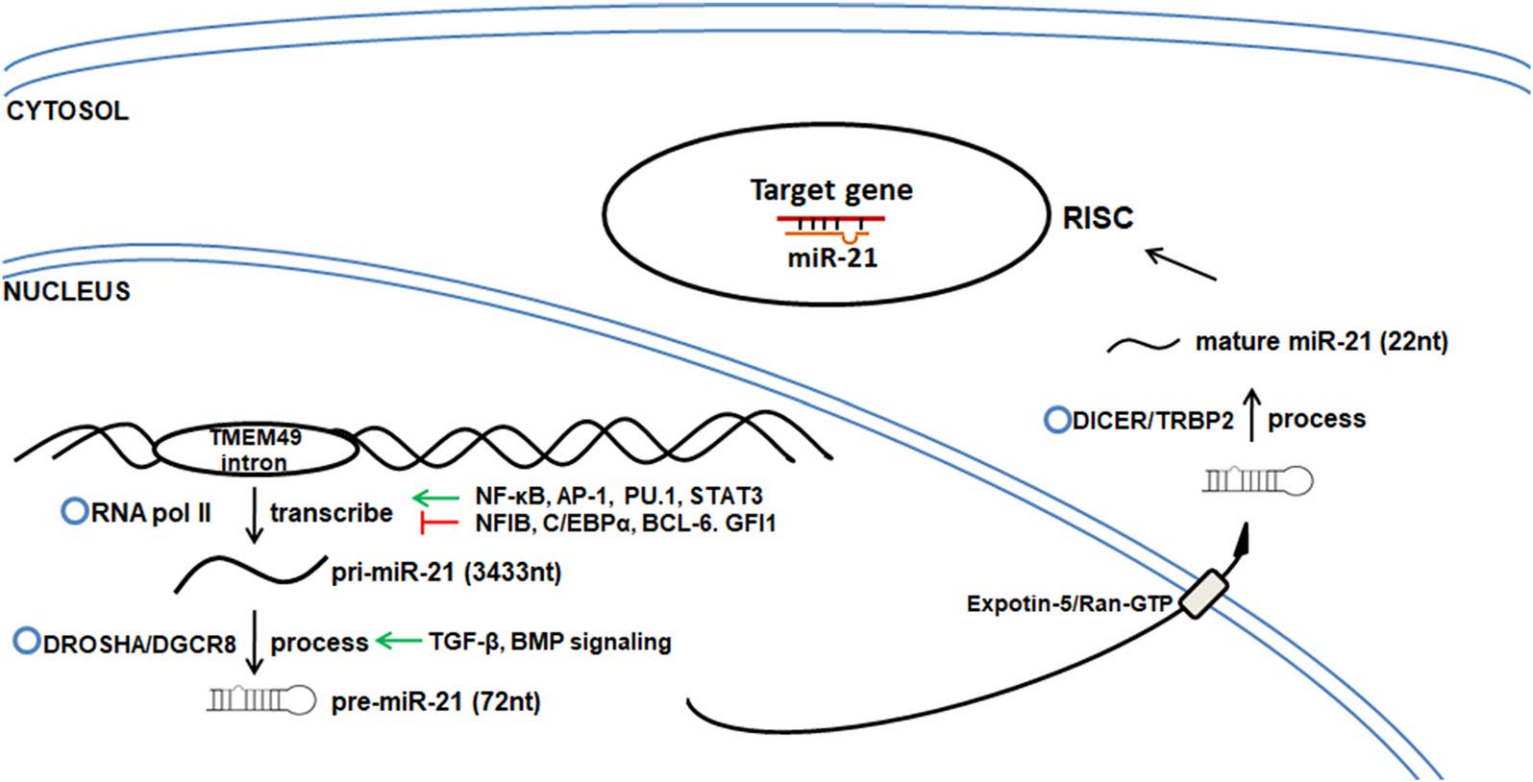
The process of miR-21 transcription and biogenesis

As we mentioned above, there are multiple players involved during the transcription and biogenesis of miR-21 (Fig. 1). Based on such understanding, we would like to discuss the possible upstream signaling for miR-21 regulation in pulmonary remodeling by following various clues extracted from previous reports.

### Possible upstream signaling for miR-21 regulation during pulmonary remodeling

#### Transforming growth factor β (TGF-β) and bone morphogenetic protein (BMP) signaling

TGF and BMP super-family share the similar signal transduction, with sequential activation of two serine-threonine kinase receptors and receptor-specific transcription factor (Smads). Both signaling pathways are crucial for lung vasculature remodeling [37]. TGF-β and BMP induce a contractile phenotype in human vascular smooth muscle cells (VSMC) through a SMAD dependent pathway and mediate a post-transcriptional activation of miR-21 by processing pri-miR-21 to pre-miR-21 [36]. The miR-21 inhibitor could block the TGF-β/miR-21/PDCD4 pathway [38]. miR-21, activated by BMP4, can target multiple DOCK proteins and modulate the activity of Ras 1 small GTPase to promote vascular smooth muscle contractility in pulmonary artery smooth muscle cells (PASMC) [39].

#### Epidermal growth factor receptor (EGFR) signaling

EGFR gene is associated with airway hyper-reactivity (AHR) in patients [40]. EGFR activation (EGFR phosphorylation at Tyr 1173) is found in lungs, and bronchiolar epithelial cells of house dust mite (HDM) induced asthma mice, while its inhibition can reduce AHR, airway smooth muscle cell thickening and goblet cell metaplasia during HDM treatment, suggesting EGFR in airway epithelium plays an essential role in mediating AHR and lung remodeling in a chronic allergic asthma model [41]. EGFR gene mutations, which lead to the insensitivity of EGFR-tyrosine kinase inhibitors (EGFR-TKI) are prevalent in never-smoker with lung cancers. Aberrant up-regulation of miR-21 in lung carcinoma cell is found significantly correlated with phosphorylated-EGFR (p-EGFR) [42].

#### Notch signaling

Notch signaling pathway plays a crucial role in angiogenesis and vascular remodeling. mRNA expressions of Notch 1 receptor and downstream factors are induced with a peak at 1–2 weeks in pulmonary vascular remodeling of rats with pulmonary hepertension [43]. Its signaling activation also facilitates the morphological, phenotypic and functional changes consistent with endothelial-to-mesenchymal transformation in HMEC-1 microvascular endothelial cells [44]. Over-expression of NOTCH1 leads to the induction of miR-21 expression as well as EMT phenotype in AsPC-1 pancreatic cancer cells [45].

These observations indicated that transcriptional regulation on miR-21 could be an important mechanism to understand the role of TGF, BMP, EGFR and Notch signaling during pulmonary remodeling.

### The validated miR-21 targets participate in various events of pulmonary remodeling

**Fig. 2:**
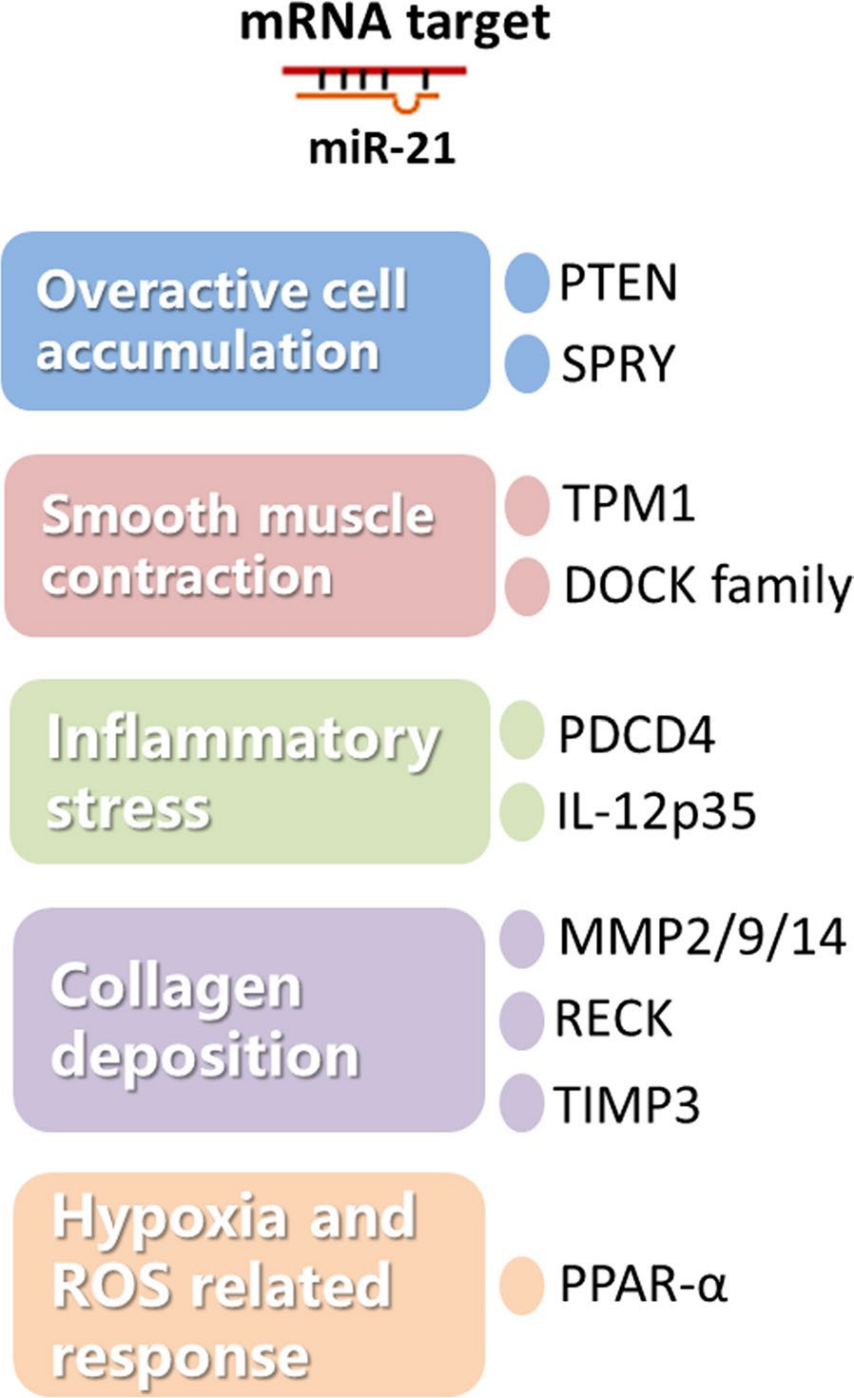
The validated miR-21 targets participating in various events of pulmonary remodeling

Till now, mounting putative miR-21 targets have been validated based on experimental evidence. Among them, several targets also play their role in multiple events of pulmonary remodeling (Fig. 2), e.g., the overactive cell accumulation, smooth muscle contraction, inflammatory stress (trigger for lung epithelium damage), extracellular matrix deposition and hypoxia-induced disorders.

#### Targets in overactive cell accumulation

Phosphatase and tensin homolog (PTEN), a phosphatidylinositol-3,4,5-trisphosphate 3 (PIP_3_)-phosphatase that inhibits phosphoinositide-3-kinase (PI3K) pathway, blocks AKT (also known as protein kinase B) signaling activation and hence acts as a tumor suppressor. PTEN is initially identified as a miR-21 target in Mz-ChA-1 human cholangiocarcinoma cells [46]. Besides, the gain or loss of miR-21 function was found to mediate the proliferation of human airway smooth muscle cell through the PI3K pathway by target regulating the PTEN protein [47].

Sprouty homolog (spry) suppresses proliferation through extracellular response kinase (ERK)-mitogen activated protein kinase (MAPK) signaling. Spry 1 and 2 are expressed both in epithelial and peripheral mesenchymal cells of the lung during embryonic development [48]. Negative regulation on SPRY1 by miR-21 is found in TGF-β mediated EMT, in which SPRY1 inhibition leads to a smaller size of the scratch wound by limiting cell motility [49].

As we mentioned, among multiple validated miR-21 targets, the PTEN and SPRY were considered as crucial players in regulating hyperproliferation during different pathogenesis. Their involvement might help us understand the role of miR-21 during overactive fibroblast accumulation.

#### Targets in muscle contraction

Tropomyosin 1 (TPM1), a rod-like helical protein that dimerizes and binds to actin, controls smooth muscle contraction and relaxation, and thus has a major role in the regulation of cell shape and function [50]. It is identified as a miR-21 target in MCF-7 human breast cancer cells [51]. Dedicator of cytokines (DOCK) protein family is mostly guanine nucleotide exchange factors for Rac and Cdc42. Multiple DOCK members are identified as miR-21 targets by base pairing at sites other than the seed region. This target inhibition of DOCK family by miR-21 leads to pulmonary vascular smooth muscle cell (PVSMC) contractility [39]. Hence, TPM1 and DOCK target inhibition might contribute to the role of miR-21 in muscle contraction dysfunctions during pulmonary remodeling.

#### Targets related to inflammatory stress

Lung epithelium damage induced by inflammatory stress is a major trigger for pulmonary remodeling. As we know, during this process, the skewed Th1/Th2 balance is entirely responsible. Interleukin-12 (IL-12) is a heterodimer composed of the 35kD subunit named IL-12p35 (IL-12A) and another 40kD subunit. IL-12 is required for T-cell-independent IFN-γ induction. Hence it is considered as a molecule germane to Th1 cell polarization. Recently, IL-12p35 is identified as a direct miR-21 target, and its expression reduction is found in asthmatic mice lungs [52].

Programmed cell death 4 (PDCD4) promotes apoptosis and positively regulates E-Cadherin and tissue inhibitor of metalloproteinase 2 (TIMP2) [3]. Three SNPs in the PDCD4 gene were found significantly associated with severe childhood asthma [53]. The expression of miR-21 RNA (detected by using in situ hybridization) and its validated target PDCD4 [54, 55] protein (detected by using immunostaining) shows a mutually exclusive pattern in colorectal epithelial cells [56]. PDCD4, as an apoptosis marker, was found to play a proinflammatory role which promotes NF-κB and suppresses the IL-10 [57]. Our previous study also discovered that its overexpression could lead to the macrophage alternative activation and airway remodeling during allergic pulmonary inflammation [58].

It was possible that the dysregulated miR-21 could repress its target IL-12p35 and PDCD4 hence contribute to an imbalanced Th1/Th2 and M1/M2 polarization and the inflammatory stress driven epithelium damage, which eventually leads to the pulmonary remodeling.

#### Targets in extracellular matrix deposition

Matrix metalloproteinase (MMPs) related tissue damage is crucial in extracellular matrix deposition. Reversion-inducing-cysteine-rich protein with Kazal motifs (RECK) participates in tumor metastasis and angiogenesis by modulating matrix metalloproteases (MMPs) including MMP 2, 9, 14, and is validated as a target of miR-21 in gastric cancer cells [59]. RECK is found down-regulated by IL-13 in the lungs from the asthmatic mice [52]. MMP-9 is present in low quantities in the healthy adult lung but highly abundant in asthmatic condition. Both the inflammatory cells and intrinsic lung cells such as epithelial cells can produce MMP-9 [60]. Both RECK (membrane-anchored matrix MMP inhibitors) and tissue inhibitor of metalloproteinase (TIMP3) gene, suppressors of malignancy and inhibitors of MMPs are found to be targets of miR-21. RECK and TIMP3 were suppressed by miR-21 and lead to MMPs induction as well as cancer cell invasion in Hela and A172 cell lines [61]. Hence, RECK and TIMP3 inhibition could be a potential mechanism to help understand the role of miR-21 in extracellular matrix deposition.

#### Targets in hypoxia-induced disorders

Peroxisome proliferator-activated receptor-α (PPAR-α), a nuclear receptor acts as a translational factor which is involved in cell proliferation, cell differentiation and inflammation responses, is identified as a miR-21 target, and may participate in hypoxia-induced PASMC proliferation [62]. More interestingly, attenuated PPAR-α expression by miR-21 targeting is found to augment AP-1 mediated miR-21 overexpression in an autoregulatory loop [63]. miR-21 might play a role in hypoxia-induced disorders through regulating its target PPAR-α.

### Dysregulated miR-21 expression profile in pulmonary remodeling related events

Previously, miR-21 was found to strongly implicated in fibrosis of various tissues including lung [64]. In this work, the dysregulated miR-21 expression in various aspects of pulmonary remodeling was discussed in detail.

#### Dysregulated miR-21 in inflammatory stress-driven epithelium damage

Alveolar epithelium plays a vital role in maintaining lung homeostasis. However, inflammatory stress and physical injury may cause epithelium damage and hence trigger airway remodeling [65]. The skewed balance between Th1 and Th2 cytokines plays an important role in inflammatory disorders causing lung remodeling such as asthma. miR-21 is found to regulate Th2-driven inflammation and inflammatory cells infiltration stress via promoting Th2 type immune response in lungs. miR-21 increases sharply (p < 0.05) in the airway wall of house dust mite-induced asthmatic mice in contrast with control [66]. miR-21 is also up-regulated in lungs of deoxycycline-induced lung-specific IL-13 transgenic mice (with allergic airway inflammation), A. fumigatus allergen-induced allergic airway inflammation and IL-4 lung transgenic mice [52]. As we mentioned before, miR-21 was identified to negatively target IL-12p35 (a molecule germane to Th1 cell polarization) in asthmatic mice. Overexpression of miR-21 promotes Th2 differentiation in non-polarized T cells, with increased Gata3 and decreased IL-12α expression [27]. Ablation of miR-21 in mice reduces lung eosinophilia after allergen challenge and significantly increases the levels of Th1 cytokine IFN-γ in vivo. miR-21 deficient dendritic cells also produce more IL-12 after LPS stimulation, and ovalbumin-challenged CD4^+^ T cells produce more IFN-γ and less IL-4. The evidence demonstrates that miR-21 might act as a major regulator of Th1 vs. Th2 responses [67].

#### Dysregulated miR-21 and EMT transition

In tissue remodeling, fibroblast is very likely to derive from epithelial or endothelial cells via epithelial-to-mesenchymal transition (EMT) or endothelial-to-mesenchymal transition (EndMT). During lung remodeling, the contribution of alveolar epithelial cells (AECs) to effective myofibroblast through EMT process has also been well acknowledged [68]. TGF-β signaling can potently induce this EMT or EndMT [69]. TGF-β signaling is mediated by the heterotetrameric complex of the trans-membrane receptor of TGFβRI and TGFβRII. After ligand activation, the SMAD2 and SMAD3 are phosphorylated by TGFβRI, bind to cytoplasmic Smad4, shuttle into the nucleus and eventually induce TGF-β expression. During cardiac injury or TGF-β stimulation, EMCs undergo EMT transition and promote both a fibroblast-like phenotype and an ectopic expression of up-regulated miR-21, while anti-miR-21 treatment can block this effect [49]. Besides, miR-21-3p, the passenger strand of mature miR-21 (miR-21-5p) was also found overexpressed in the TGF-β_1_ induced primary parenchymal lung fibroblasts based on microarray data and further validated using RT-qPCR [70]. For now, the conclusion that TGF-β induced pre-miR-21 overexpression in the EMT process during tissue fibrosis was widely accepted. However, to find out which strand from this precursor (miR-21-5p, miR-21-3p or both) plays a more prominent role in this process still needs further validation.

#### Dysregulated miR-21 in the transdifferentiation of fibroblasts into myofibroblast

The transdifferentiation of fibroblasts into myofibroblasts (MFs) plays an essential part in tissue remodeling. Pathological remodeling can be induced by TGF-β cytokine or triggered by injury. In vascular remodeling, miR-21 is found to be overexpressed in MFs with a 4.8-fold change against adventitial fibroblasts (AFs) expression. Overexpression of miR-21 by using pre-miR-21 treatment increases the proliferation and decreases apoptosis of AFs and MFs. Furthermore, miR-21 inhibition can reverse the vascular remodeling induced by balloon injury [71]. In cardiac remodeling, abnormal up-regulation of miR-21 targets SPRY1 and hence augments the ERK-MAPK signaling in cardiac fibroblast, which leads to fibroblast survival and its differentiation into myocyte [72].

miR-21 also participates in TGF-β_1_-induced fibroblast-to-myofibroblast transdifferentiation in cancer stroma by targeting PDCD4. miR-21 is upregulated in TGF-β and cancer medium activated fibroblasts. Gain or loss of function assay further demonstrates the effect of miR-21 on TGF-β_1_-induced MF transdifferentiation [73]. Up-regulation of miR-21 is found in the lungs of mice with bleomycin-induced fibrosis and also in the lungs of patients with idiopathic pulmonary fibrosis (IPF) [74]. Immunohistochemistry and in situ hybridization (ISH) demonstrate the miR-21 expression mainly localizes with α-SMA (a marker for MF), suggesting it as the primary source of up-regulated miR-21 in lung fibrosis. TGF-β_1_ enhances miR-21 in primary pulmonary fibroblasts, and more interestingly, this elevated miR-21 expression can amplify a circuit which eventually leads to TGF-β_1_ signaling activation by negatively targeting an inhibitory SMAD protein, the SMAD7 [74].

Here, mounting evidence seems to suggest that up-regulation of miR-21 could lead to the transdifferentiation of fibroblasts into myofibroblasts, which is the cellular signature for the EMT process during pulmonary remodeling.

#### Dysregulated miR-21 in hypoxia stimuli and ROS response

One of the most critical lung functions is to maintain adequate oxygen. Chronic hypoxia is an important trigger for pulmonary vascular remodeling. Hypoxia favors ROS and NOS production causing pulmonary oxidative stress [75]. Hypoxia can induce PASMC proliferation. Cell proliferation and miR-21 expression are found increased in human PASMC during hypoxia (3% O_2_), and miR-21 can induce proliferation in normoxia condition. Reduction of miR-21 leads to a significant decrease in hypoxia-induced cell proliferation [62]. Moreover, dysregulated miR-21 expression was also found in the pathogenesis of chronic hypoxia-induced pulmonary vascular remodeling [76].

In vascular smooth muscle cells (VSMC), hydrogen peroxide (H_2_O_2_) induces apoptosis and cell death, as well as the up-regulation of miR-21. Pre-miR-21 plays a protective role in VSMC apoptosis by targeting PDCD4 [77]. miR-21 also protects cardiac myocytes from H_2_O_2_ mediated injury [78]. In human aortic endothelial cells (HAEC), senescent phenotype leads to reduced miR-21 expression, which is positively associated with endothelial nitric oxide synthase (eNOS) activation (phosphorylation of eNOS at serine 1177) and the production of nitric oxide [79]. Nitric oxide is generally accepted as an indicator for both oxidative stress and airway inflammation. Fractional exhaled nitric oxide (FeNO) is considered as emerging biomarkers for lung inflammation and is recommended as a supplemental index to evaluate clinical interventions on airway disorders such as asthma [80].

Such evidence indicates that dysregulated miR-21 could actively participate in hypoxia stimuli and ROS response during pulmonary remodeling.

#### Dysregulated miR-21 in other pulmonary remodeling related events

In other pulmonary remodeling related events such as overactive fibroblast (myofibroblast) accumulation, extracellular matrix deposition, angiogenesis and tissue regeneration, there is also plenty of observation support that miR-21 might also play a crucial part.

In bleomycin-treated lungs (a mature lung remodeling animal model), increased miR-21 expression is primarily localized in the sheets of the lung parenchymal with fibroblast/MF accumulation [74]. miR-21 antisense in vivo treatment can attenuate enhanced extracellular matrix deposition and ECM proteins such as COL1A and COL1B after induction for 3 weeks [74]. miR-21 overexpression can induce HIF-1 and VEGF expression, the activation of AKT and ERK1/2 as well as angiogenesis in DU145 prostate cancer cells. Moreover, HIF-1α is found crucial for miR-21-upregulated angiogenesis [81]. miR-21 is also found to regulate tissue regeneration. In mice after 2/3 partial hepatectomy, there is a notable induction of miR-21 expression which directly inhibits BTG2, a cell cycle inhibitor. miR-21 induction is critical for hepatocyte proliferation during liver regeneration [82].

## Conclusion

Transcriptional regulation on miR-21 could be an essential mechanism to understand the role of TGF, BMP, EGFR and Notch signaling during pulmonary remodeling. Several miR-21 targets play their role in multiple events of pulmonary remodeling, e.g., the overactive cell accumulation, smooth muscle contraction, inflammatory stress (trigger for lung epithelium damage), extracellular matrix deposition and hypoxia-induced disorders. Dysregulated miR-21 expression profile was found in many aspects of pulmonary remodeling including inflammatory stress-driven epithelium damage, EMT transition, transdifferentiation of fibroblasts into myofibroblast, hypoxia stimuli and ROS response, as well as some other pulmonary remodeling related events.

In summary, miR-21 might play a crucial role in pulmonary remodeling, which makes it a promising candidate miRNA for further study. We tried to follow many clues for its role in pulmonary remodeling. However, the direct proof is still in need. Hence, our work calls for the experimental investigation into above-mentioned aspects and hope such novel clues could be quite useful for further research in this area.

## Data Availability

Not applicable.

## Abbreviations

AHR: airway hyperreactivity
BALF: bronchoalveolar lavage fluid
COPD: chronic obstructive pulmonary disease
EMCs: epicardial mesothelial cells
EMT: epithelial-to-mesenchymal transition
EndMT: endothelial-to-mesenchymal transition
FeNO: fractional exhaled nitric oxide
HAEC: human aortic endothelial cells
HDM: house dust mite
IPF: idiopathic pulmonary fibrosis
MFs: myofibroblasts
NO: nitric oxide
PASMC: pulmonary artery smooth muscle cells
ROS: reactive oxygen species
VSMC: vascular smooth muscle cells
